# ﻿A new *Hemicyclops* (Copepoda, Cyclopoida, Clausidiidae) associated with the scleractinian coral *Galaxea* from the South China Sea

**DOI:** 10.3897/zookeys.1260.168539

**Published:** 2025-11-18

**Authors:** Il-Hoi Kim, Jia Wang, Viatcheslav N. Ivanenko

**Affiliations:** 1 Korea Institute of Coastal Ecology, 302-802, Seokcheon-ro 397, Bucheon 14449, Republic of Korea Korea Institute of Coastal Ecology Bucheon Republic of Korea; 2 Biological Faculty, Shenzhen MSU-BIT University, Shenzhen 518172, China Shenzhen MSU-BIT University Shenzhen China; 3 Department of Invertebrate Zoology, Lomonosov Moscow State University, Moscow 119992, Russia Lomonosov Moscow State University Moscow Russia

**Keywords:** Association, Copepoda, coral host, Crustacea, new species, Scleractinia, symbiosis, taxonomy

## Abstract

The genus *Hemicyclops* Boeck, 1873 is known for its association with various marine invertebrates, including cnidarians, crustaceans, polychaetes, and sponges, with some species also occurring in planktonic communities. Here, we report the first association of *Hemicyclops* with the scleractinian coral *Galaxea fascicularis* (Linnaeus, 1767) (Scleractinia, Euphylliidae). *Hemicyclops cyanus***sp. nov.** is described based on a female specimen collected from this coral host in the lagoon (depth 10 m) of Dongsha Atoll, Pratas Islands, South China Sea. The new species is readily distinguished from its congeners by its characteristic genital double-somite, which bears prominent anterolateral expansions, and by the flexed, elongated exopodal segment of leg 5, which is more than three times longer than wide. In *H. cyanus***sp. nov.**, the paired spermatophores attached to the female are fused into a butterfly-shaped, highly modified complex with large lateral wings and a central tube into which the female urosome is inserted. To aid in species identification, we present the first comparative plate with schematic illustrations of the genital double-somites for the group of 25 species, including the type species.

## ﻿Introduction

Copepods are among the most diverse and ecologically significant crustaceans in the ocean, with numerous species forming symbiotic associations with scleractinian corals (Humes 1985a; Korzhavina and Ivanenko 2021). Based on at least 1195 documented records, these associations involve at least 384 species of copepods belonging to 117 genera and 29 families, and 172 species of corals belonging to 67 genera and 16 families (Korzhavina and Ivanenko 2021). Despite the high diversity and abundance of these copepods, only a fraction of potential coral hosts has been studied, and most research has been limited to a few geographic regions, leaving major gaps in knowledge of their taxonomy, host specificity, and biogeography.

The Indo-Pacific coral genus *Galaxea* Oken, 1815 (Scleractinia Bourne, 1900; Euphylliidae Milne Edwards & Haime, 1857) is an important reef-builder and a host for at least 18 copepod species in eight genera, belonging to the orders Cyclopoida Burmeister, 1834 and Siphonostomatoida Burmeister, 1835 (Humes 1979, 1985a, b, 1991, 1996; Cheng et al. 2016; Cheng and Dai 2018). Cyclopoida includes 11 species of Anchimolgidae Humes & Boxshall, 1996, two species of Xarifiidae Humes, 1960, and one species of Pterinopsyllidae Sars, 1913. Siphonostomatoida includes four species of Asterocheridae Giesbrecht, 1899. Most copepod records come from *Galaxea fascicularis* (Linnaeus, 1767), followed by *G. astreata* (Lamarck, 1816), with occasional finds on *G. horrescens* (Dana, 1846) or unidentified *Galaxea* species.

These copepod communities occur in many parts of the Indo-Pacific, including the Moluccas, New Caledonia, Australia, Madagascar, and Dongsha Atoll in the northern South China Sea. Dongsha Atoll is a biodiversity hotspot for *Galaxea*-associated copepods, with eight recorded species, two of which were first described from this atoll by Cheng and Dai (2018). The atoll also serves as a stepping-stone for larval dispersal and genetic connectivity among coral reefs in the South China Sea (Liu et al. 2021) and may function as a thermal refuge for reef-building corals under climate change (Tkachenko and Soong 2017). However, repeated disturbances such as typhoons, mass bleaching, and human activities have reduced coral cover, raising concerns about long-term reef decline (Cheng et al. 2020).

Here, we describe a new species of *Hemicyclops* Boeck, 1873 (Copepoda, Cyclopoida, Clausidiidae Embleton, 1901) found on the scleractinian coral *Galaxea*, as part of a broader study on the biodiversity of coral-associated copepods.

## ﻿Material and methods

Symbiotic copepods were collected from colonies of *Galaxea fascicularis* at the Dongsha Atoll, Pratas Islands, South China Sea (see Suppl. material 1: figs S1–S5), using a boat and SCUBA diving, applying the method described by Ivanenko et al. (2018). Coral specimens were obtained from depths of 10 m and placed into resealable plastic bags underwater. Upon retrieval to the surface, each bag with corals was filled with a 10% ethanol solution to preserve the associated fauna. After approximately 30 minutes, the bags were gently agitated, and the preservation fluid was filtered through a 100 μm mesh sieve. The retained material was transferred to a Petri dish for the selection of copepods under a stereomicroscope. Selected specimens were preserved in 95% ethanol and stored at −20 °C.

Prior to microscopic examination, specimens were cleared in lactic acid for approximately 10 minutes. Dissections followed the reverse slide method described by Humes and Gooding (1964). Appendage lengths were measured, with reported values representing the mean of the maximum and minimum measurements. The holotype is deposited in the Honam National Institute of Biological Resources (HNIBR).

## ﻿Taxonomy

**Order Cyclopoida Burmeister, 1834**

**Family Clausidiidae Embleton, 1901**

**Genus *Hemicyclops* Boeck, 1873**

### ﻿ Hemicyclops cyanus sp. nov.

Taxon classificationAnimaliaCyclopoidaClausidiidae

C3CB7246-CF55-5527-A094-1F366EB27830

https://zoobank.org/0C5566E0-E628-4749-A9C2-4DA06FB2943F

Figs 1, 2, 3

#### Type material.

***Holotype***: One dissected ♀ mounted on 3 slides (HNIBRIV18791).

#### Type locality.

20°38'03.1"N, 116°49'30.5"E, Lagoon of Dongsha Atoll, Pratas Islands, the South China Sea, depth 10 m, 12 October 2017. Collector: V.N. Ivanenko.

#### Host.

*Galaxea fascicularis* (Linnaeus, 1767) (Scleractinia, Euphylliidae).

#### Etymology.

The name is derived from the Greek “cyan” (meaning “blue-green”), referring to the blue-green colour of the holotype.

#### Description.

**Figure 1.**
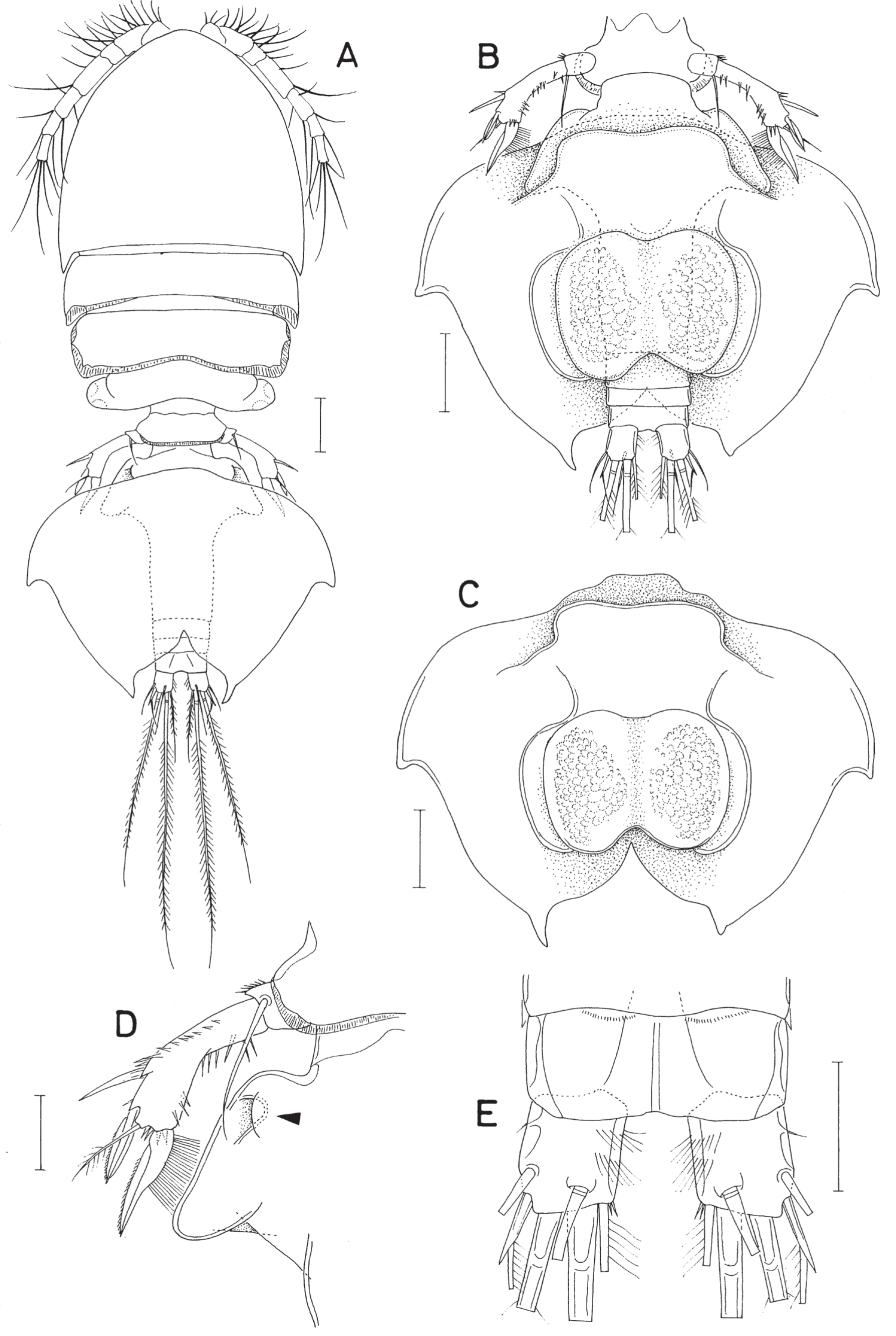
*Hemicyclops cyanus* sp. nov., female. A. Habitus with spermatophore; B. Urosome with spermatophore, ventral; C. Spermatophore; D. Left side of anterior part of urosome, dorsal; E. Anal somite and caudal rami, dorsal. Scale bars: 0.1 mm (A–C); 0.05 mm (D, E).

**Female.** Body (Fig. 1A) rather narrow, with a vivid blue-green colour all over. Prosome 732 × 477 μm, consisting of the cephalothorax and second to fourth pedigerous somites, with nearly parallel lateral margins. Cephalothorax 436 μm long, wider than long, with projected and acutely pointed posterolateral corners. The second and third pedigerous somites bearing a membranous fringe along their posterodorsal margin.

**Figure 2.**
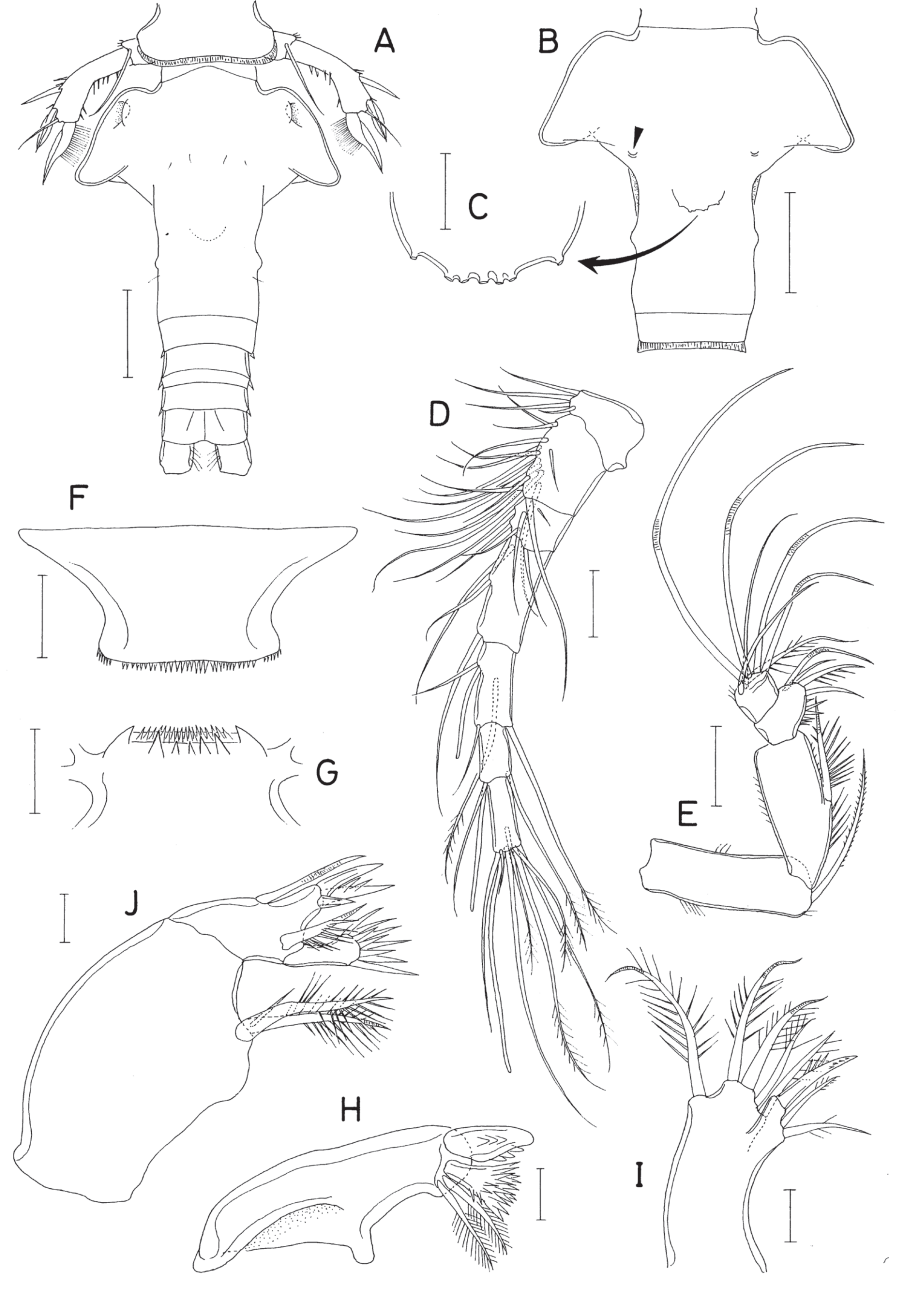
*Hemicyclops cyanus* sp. nov., female. A. Urosome, dorsal; B. Genital double-somite, ventral; C. Ventral organ of genital double-somite; D. Antennule; E. Antenna; F. Labrum; G. Labium; H. Mandible; I. Maxillule; J. Maxilla. Scale bars: 0.1 mm (A, B); 0.02 mm (C, H–J); 0.05 mm (D–G).

Urosome (Figs 1B, 2A) 5-segmented. First urosomal somite (fifth pedigerous somite) 163 μm wide, with a membranous fringe along the posterodorsal margin. The genital double-somite 1.08 times longer than wide (330 × 309 μm); its anterior 40% wing-like, expanded laterally, tapering towards posterolaterally, with a blunt apex. Genital aperture (Fig. 1D; indicated by arrowhead) positioned dorsolaterally at the proximal region of the expanded part. Each posterolateral region of this expanded part bearing a small copulatory pore (Fig. 2B; indicated by arrowhead). The narrower posterior 60% part of the double-somite bearing 1 small tubercle on each lateral margin and 1 plate-like ventral organ (Fig. 2B) on the anteroventral region, with 7 pores along the distal margin. The genital double-somite and three abdominal somites with a membranous fringe along their posteroventral margin. The three abdominal somites are short, measuring 45 × 106, 30 × 103, and 36 × 102 μm, respectively. The anal somite bears a large anal aperture.

Spermatophore complex (Fig. 1B, C), formed by the fusion of the original pair of spermatophores, is strongly modified, with a tube-like medial region encircling the urosome and wing-like lateral expansions, each bearing lateral and posterior apices. Each spermatophore internally contains bubble-like transparent globules of irregular sizes; a space is present lateral to the spermatophore.

Caudal rami (Fig. 1E) widely separated from each other; each ramus 1.15 times longer than wide (45 × 39 μm), bearing two transverse rows of fine setules on the inner side, one minute proximal setule (rudimentary seta I) on the outer margin, and six setae (setae II–VII). Seta IV (outer distal seta) proximally modified as a spine tipped with a unilaterally pinnate seta.

Rostrum small, triangular, with a blunt posterior apex.

Antennule (Fig. 2D) 354 μm long, 7-segmented; armature formula: 4, 14, 6, 3, 4+aesthetasc, 2+aesthetasc, and 7+aesthetasc; fifth to terminal segments each with 2, 1, and 4 pinnate setae, respectively; all other setae naked; aesthetascs thin, setiform.

Antenna (Fig. 2E) 4-segmented, consisting of the coxobasis and 3-segmented endopod; coxobasis with 1 large seta at the mediodistal region, 3 patches of several setules on the margins; first endopodal segment slightly shorter than coxobasis, bearing 1 spinulose seta on the inner margin and setules or spinules on both margins; second endopodal segment expanded mediodistally (but lacking a digitiform projection), armed with 4 setae (the second proximal seta ornamented with spinules along the inner margin) and several spinules on the inner margin; third endopodal segment wider than long, 24 × 27 μm, subquadrate, bearing 7 setae, one of them spinulose, the others (including 4 geniculated ones) naked.

Labrum (Fig. 2F) broad, with a row of spinules along the posterior margin.

Labium (Fig. 2G) bearing a row of fine spinules along the anterior margin, 1 tooth at each side of the anterior margin, and 5 spinules on the ventral surface near the anterior margin.

Mandible (Fig. 2H) armed distally with 1 stout spine bearing 2 rows of 3 denticles, 1 flabelliform spine, and 2 pinnate setae.

Maxillule (Fig. 2I) distally bilobed; smaller inner lobe with 3 setae, one of them spiniform; larger outer lobe with 5 pinnate setae.

Maxilla (Fig. 2J) 2-segmented; syncoxa (proximal segment) with 2 spinulose, spiniform setae of equal length, one of them bearing 1 small subsidiary seta at the proximal region; basis (distal segment) with 1 naked ventral seta, distal projection tipped with 3 unequal spines (one of them bearing 2 spinules), 1 spinulose seta, and 1 large, flabelliform spine.

**Figure 3.**
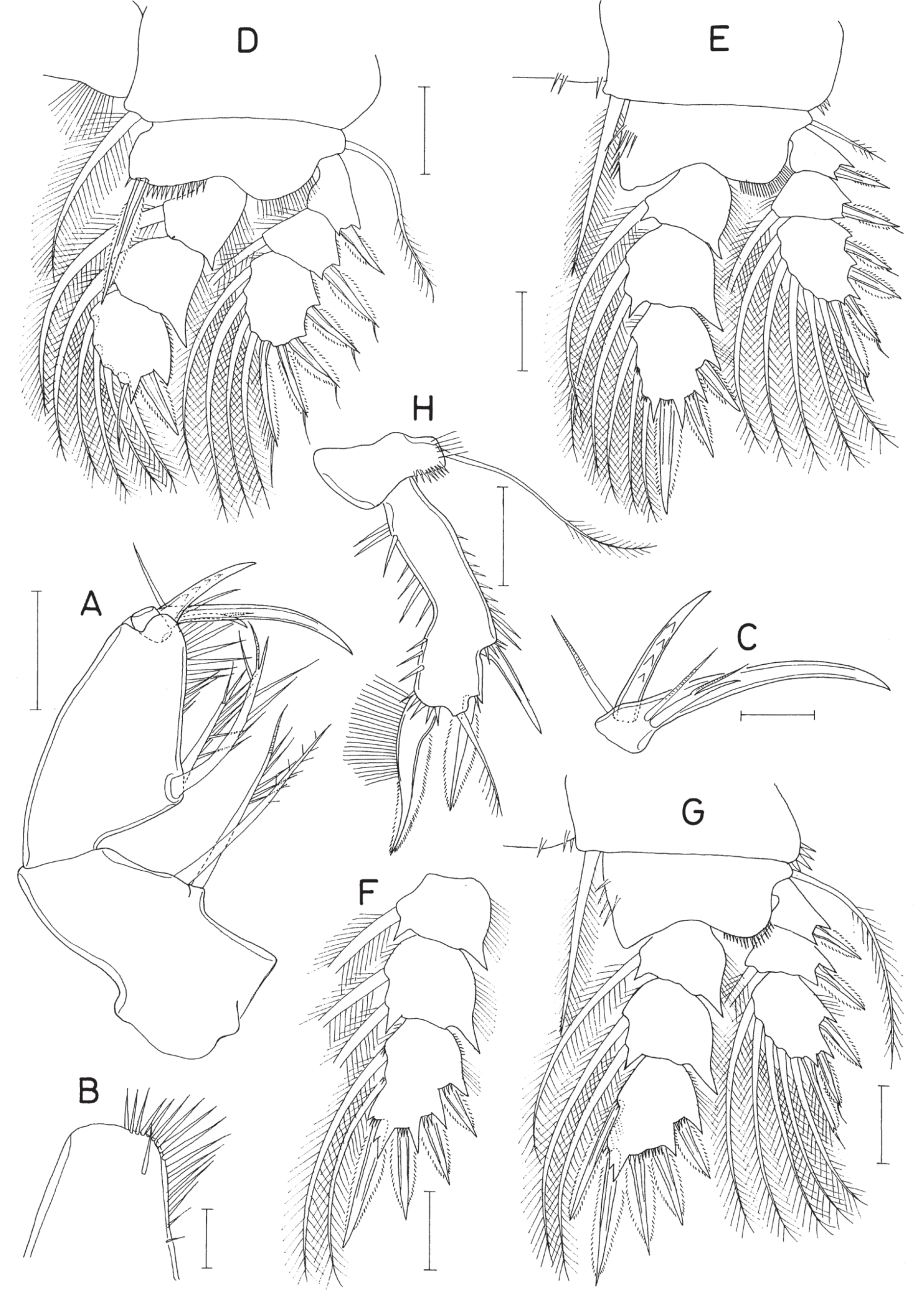
*Hemicyclops cyanus* sp. nov., female. A. Maxilliped; B. Distal part of maxilliped basis; C. Terminal segment of maxilliped; D. Leg 1; E. Leg 2; F. Endopod of leg 3; G. Leg 4; H. Leg 5. Scale bars: 0.05 mm (A, D–H); 0.02 mm (B, C).

Maxilliped (Fig. 3A) consisting of the syncoxa, basis, and 2-segmented endopod; syncoxa (first segment) with 2 large, equal setae, one of them feebly pinnate, the other bearing several spinules; basis (second segment) longest, armed with 2 large spinulose setae at the apex of the projected proximal third and ornamented with about 17 setae at the mediodistal region, one of these spinules separated from the others (Fig. 3B); first endopodal segment (third segment) small, unarmed; second endopodal segment (Fig. 3C) forming a long claw, proximally bearing 1 strong spine bearing 4 denticles, 2 naked setae, and 1 small spine bearing 1 setule subdistally.

Legs 1 (Fig. 3D), 2 (Fig. 3E), 3, and 4 (Fig. 3G) biramous, with 3-segmented rami. The intercoxal plate bearing setules in leg 1 and spinules in legs 2–4. The inner coxal seta is large, stiff, and spiniform in legs 2–4. Leg 3 is similar to leg 2, except bearing 4 spines and 2 setae on the third endopodal segment (Fig. 3F). The outer seta on the basis is large in legs 1 and 4, but small in legs 2 and 3. The mid-distal margin of the basis is ornamented with setules in leg 1 but with spinules in legs 2–4. The inner distal spine on the basis of leg 1 is large, extending to the distal margin of the second endopodal segment. The inner distal margin of the basis of leg 1 has a row of spinules near the base of the inner distal spine. The inner surface of the basis of legs 2–4 bears several spinules. The inner distal process of the third endopodal segment of legs 2–4 is unequally bifurcated. The distal seta on the third endopodal segment of leg 1 is small and naked. The distal flagellum is found on 5 distal spines in leg 1 and 2 distal spines in legs 2–4.

Armature formula for legs 1-4 as follows:

**Table:** 

	Coxa	Basis	Exopod	Endopod
Leg 1	0-1	1-I	I-0; I-1; III, I, 4	0-1; 0-1; I, 5
Leg 2	0-1	1-0	I-0; I-1; III, I, 5	0-1; 0-2; I, II, 3
Leg 3	0-1	1-0	I-0; I-1; III, I, 5	0-1; 0-2; I, II, I+2
Leg 4	0-1	1-0	I-0; I-1; II, I, 5	0-1; 0-2; I, II, II

Leg 5 (Figs 1D, 3H) 2-segmented; protopod (proximal segment) wider than long, articulated from somite, armed with 1 long, slender, pinnate distodorsal seta and ornamented with setules and spinules near the base of the seta; exopodal segment 3.37 times longer than wide (118 × 35 μm), curved near the middle, armed with 3 spines and 1 pinnate seta, and ornamented with spinules along the outer and inner margins. The spine on the outer margin is naked, 51 μm long; the outer distal spine is 58 μm long; the distal seta is 55 μm long; the large inner distal spine is 82 μm long, slightly curved, bearing long, brush-like setules along approximately the proximal half of the inner margin.

Leg 6 not visible in dorsal view of genital aperture (Fig. 2D).

**Male.** Unknown.

## ﻿Discussion

The new species was found on *Galaxea fascicularis* and is the first record of *Hemicyclops* from the coral family Euphylliidae. *Hemicyclops* is a diverse genus with 50 known species (Walter and Boxshall 2025). These copepods are reported from many hosts, often living in their burrows, and have been found on sponges, cnidarians, crustaceans, polychaetes, and mollusks, as well as in plankton. Records from scleractinian corals are rare, with only two known cases: *H. columnaris* Humes, 1984 from *Porites lobata* Dana, 1846 (Poritidae Gray, 1840) in Panama, and *H. apiculus* Humes, 1995 from *Favia* De Blainville, 1820 (Faviidae Milne Edwards & Haime, 1857) and *Dendrophyllia* de Blainville, 1830 (Dendrophylliidae Gray, 1847) in Madagascar (Humes 1984, 1995). The present finding adds Euphylliidae to the host range of the genus. As only one specimen was collected, it is unclear whether this species is an obligate associate of *Galaxea* or another invertebrate living in the coral tissues or skeleton.

After analyzing the diagnostic features of existing *Hemicyclops* species, we found that eight species were insufficiently described, and the remaining 42 can be divided into Groups I and II. Group I includes atypical species (*N* = 17) with distinctive morphological traits that differ from the typical morphology of the genus. Group II consists of typical species (sensu stricto group) (*N* = 25) that share with *H. cyanus* sp. nov. the following core diagnostic features of the genus and serve as a reference for species identification: (1) the urosome is 5-segmented in the female and 6-segmented in the male; (2) the antennule is 7-segmented and bears four setae on the first segment, with no specified seta on the second; (3) the second endopodal segment (third segment) of the antenna bears four setae and lacks a claw; (4) the syncoxa (first segment) of the male maxilliped bears a single seta; (5) the basis of male leg 1 lacks an inner distal spine; and (6) the swimming legs have the usual number of armature elements (armature elements on the exopod and endopod of legs 1–4 are 8 and 6 in leg 1; 9 and 6 in legs 2 and 3; and 8 and 5 in leg 4, respectively).

### ﻿Group I

The following 17 species possess unusual morphological features in the female that clearly distinguish them from *H. cyanus* sp. nov. and other congeners:

1. *H. acanthophorus* Humes, 1995 — second endopodal segment of the antenna with 1 claw and 3 setae; labrum modified with digitiform lateral lobes; third exopodal segment of leg 3 with 8 (not 9) elements (Humes 1995).

2. *H. acanthosquillae* Humes, 1965 — second endopodal segment of the antenna with an elongated mediodistal projection more than twice as long as wide (Humes 1965).

3. *H. cornutus* Kim & Hong, 2014 — fifth pedigerous somite with a posterolateral hook (Kim and Hong 2014).

4. *H. ctenidis* Ho & Kim, 1990 — antennule 6-segmented; second endopodal segment of leg 4 with a single inner seta (Ho and Kim 1990).

5. *H. cylindraceus* (Pelseneer, 1929) — first antennular segment with 5 setae; second endopodal segment of the antenna with 2 claws and 2 setae; female urosome 6-segmented; terminal claw of the female maxilliped elongated, as in the male (Stock 1954).

6. *H. diremptus* Humes, 1965 — genital double-somite divided by a transverse suture line (Humes 1965).

7. *H. livingstoni* (Scott T, 1894) — first antennular segment with 5 setae; second endopodal segment of the antenna with 2 claws and 2 setae; female maxilliped with a large terminal claw, as in the male (Scott 1894).

8. *H. membranatus* Moon & Kim, 2010 — first antennular segment with 5 setae; second endopodal segment of the antenna with 1 claw and 3 setae (Moon and Kim 2010).

9. *H. nasutus* Moon & Kim, 2010 — first antennular segment with 5 setae (Moon and Kim 2010).

10. *H. nichollsi* Karanovic, 2008 — first antennular segment with 5 setae (Karanovic 2008).

11. *H. perinsignis* Humes, 1973 — first antennular segment with 5 setae; second endopodal segment of the antenna with 1 claw and 3 setae; third exopodal segment of leg 4 with 9 (not 8) elements (Humes 1973).

12. *H. rapax* Lee, Chang & Kim, 2022 — female maxilliped of the male type, with a large terminal claw (Lee et al. 2022).

13. *H. sebastiani* Kihara & Rocha, 1993 — first antennular segment with 5 setae; second segment of the antennule with 1 enlarged seta at the posterodistal corner; third endopodal segment of leg 1 with 1 spine and 4 setae (Kihara and Rocha 1993).

14. *H. spinulosus* Itoh & Nishida, 1998 — first antennular segment with 5 setae (Itoh and Nishida 1998).

15. *H. tamilensis* (Thompson & A Scott, 1903) — female urosome 6-segmented (T Thompson and A Scott 1903).

16. *H. thysanotus* Wilson, 1935 — third exopodal segment of leg 4 with 9 (not 8) elements (Gooding 1960).

17. *H. vicinalis* Humes, 1995 — third exopodal segment of leg 4 with 9 elements (Humes 1995).

### ﻿Group II

Twenty-six species of *Hemicyclops* (including the type species *H. purpureus* Boeck, 1873, and *H. cyanus* sp. nov.) are listed in Table 1. It should be noted that the female urosome of *H. aberdonensis* (Scott T & Scott A, 1892) was originally illustrated as 6-segmented, but O’Reilly (2000) reported a newly collected female with a 5-segmented urosome. Therefore, *H. aberdonensis* is classified among the species of Group II. Vervoort and Ramirez (1966) considered *H. thompsoni* (Canu, 1888) to be an “insufficiently known” species, mainly because the original description lacked details and figures of the legs. However, Canu’s (1888) original illustration of the female provides several valuable taxonomic details, such as the shape of the genital double-somite, the proportional length of the caudal ramus, and the form of the antenna, in which the second endopodal segment bears a strong inner distal projection. Based on these morphological features, *H. thompsoni* should be assigned to Group II.

**Table 1.** Distinctive characters and character states of 26 species (Group II) of *Hemicyclops*.

Species	Characters 1–7*							Data sources
	1	2	3	4	5	6	7	
*H. aberdonensis* (Scott T & Scott A, 1892)	=2	<1	<1	=1.9	2	2	?	Scott T and Scott A (1892)
*H. amplicaudatus* Humes, 1965	3.55	0.86	1	3.24	1	1	X	Humes (1965)
*H. apiculus* Humes, 1995	1.08	1.28	X	1.57	–	–	–	Humes (1995)
*H. australis* Nicholls, 1944	1.0	1.35	<1	=2	1	2	+	Nicholls (1944)
*H. axiophilus* Humes, 1965	1.92	1.16	>1	1.60	1	1	X	Humes (1965)
*H. biflagellatus* Humes, 1965	2.44	1.09	>1	2.14	1	1	X	Humes (1965)
*H. columnaris* Humes, 1984	1.46	1.65	=1	2.47	1	1	X	Humes (1984)
*H. cyanus* sp. nov.	1.15	1.08	X	3.37	–	–	–	present paper
*H. gomsoensis* Ho & Kim, 1991	2.8	1.15	1	1.7	2	1	X	Kim (2015)
*H. humesi* Kim, 2009	2.75	1.79	>1	2.28	1	1	X	Kim (2009)
*H. intermedius* Ummerkutty, 1962	=1	=1.3	X	=2	–	–	–	Ummerkutty (1962)
*H. japonicus* Itoh & Nishida, 1993	1.8	=1	>1	2.37	1	1	X	Kim (2015)
*H. kombensis* Humes, 1965	2.43	1.5	>1	1.84	1	1	X	Humes (1965)
*H. magnus* Kim, 2009	2.71	1.44	>1	2.05	1	1	X	Kim (2009)
*H. mortoni* Boxshall & Humes, 1987	2.2	1	1	1.61	–	–	–	Boxshall and Humes (1988)
*H. parilis* Moon & Kim, 2010	2.69	<1	=1	1.98	2	1	X	Moon and Kim (2010)
*H. parapiculus* Kim & Hong, 2014	1.15	1.23	<1	1.80	–	–	–	Kim and Hong (2014)
*H. purpureus* Boeck, 1873	=1.5	=1.3	X	=1.8	2	1	X	Sars (1917)
*H. saxatilis* Ho & Kim, 1991	1.13	1.15	<1	1.55	1	2	+	Ho and Kim (1991)
*H. tanakai* Itoh & Nishida, 2002	2.45	1.3	=1	1.65	1	1	X	Kim (2015)
*H. thalassius* Vervoort & Ramirez, 1966	2.3	=1	1	=2	–	1	+	Vervoort and Ramirez (1966)
*H. thompsoni* (Canu, 1888)	=3	=1.3	=1.5	=1.9	–	–	–	Canu (1888)
*H. tripartitus* Kim, 2009	3.27	1.89	>1	2.06	1	1	X	Kim (2009)
*H. ventriplanus* Kim, 2000	2.55	1.13	=1	2.02	2	1	X	Kim (2015)
*H. visendus* Humes, Cressey, Gooding & 1958	1.71	1.35	>1	1.54	1	1	X	Humes et al. (1958)
*H. xiamensis* Ohtsuka, Tomikawa & Shang, 2010	=1.8	=1.1	<1	=1.6	2	1	X	Ohtsuka et al. (2010)

* Characters 1–7: 1, length/width ratio of female caudal ramus; 2, length/width ratio of female genital double-somite; 3, length/width ratio of the inner distal projection of the second endopodal segment of the antenna; 4, length/width ratio of exopodal segment of female leg 5; 5, posterolateral spines (leg 6) on male genital somite; 6, setal count on syncoxa of male maxilliped; 7, inner distal element on the basis of male leg 1. Symbols: >, more than; <, less than; =, approximately equal; +, present; X, absent; -, data missing.

**Figure 4.**
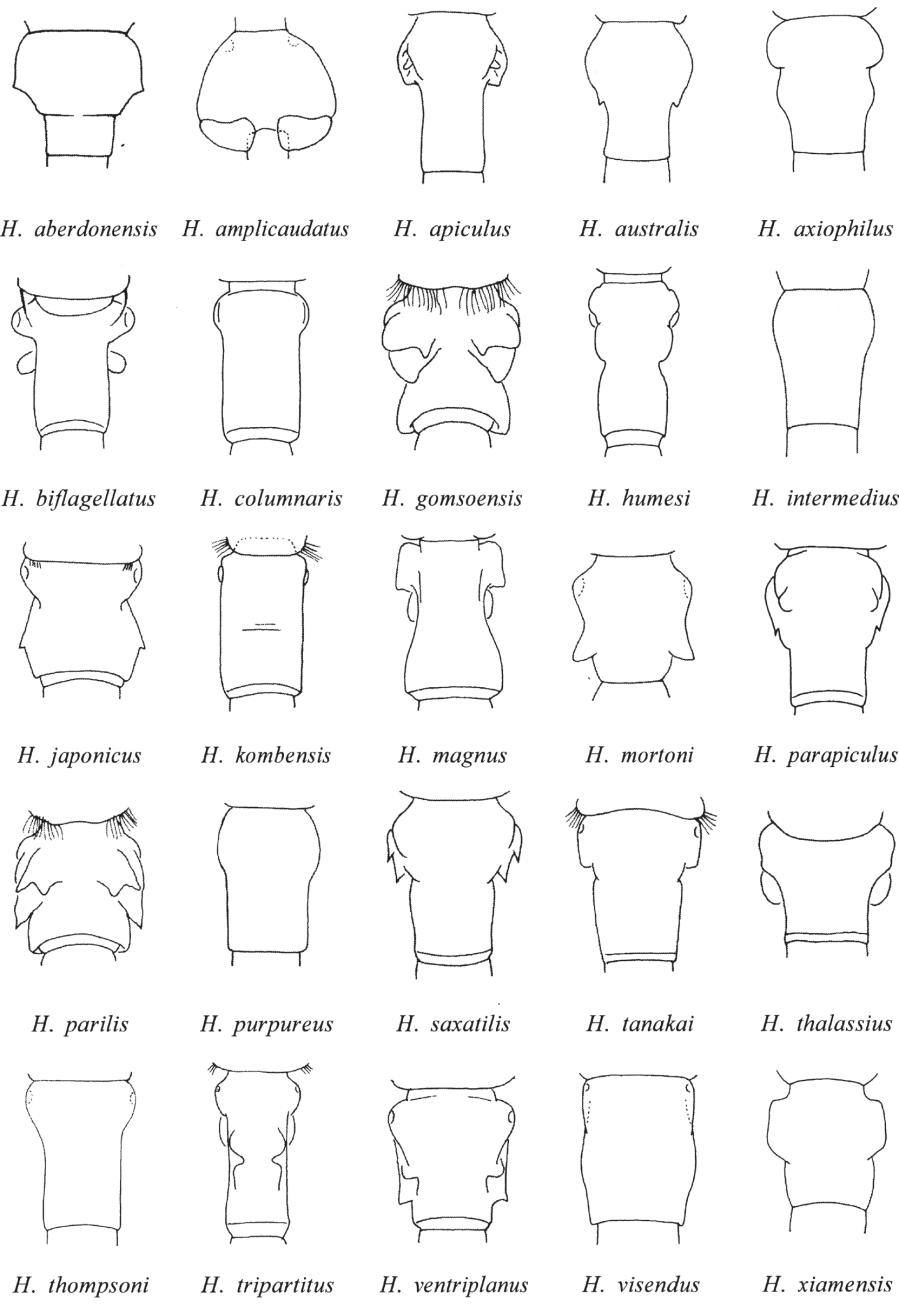
Schematic dorsal views of the female genital double-somite in 25 species of *Hemicyclops* (Group II).

Those 26 species share many morphological features and are often difficult to distinguish from one another using quantitative characters. However, they have a distinctly shaped genital double-somite (Fig. 4), which allows them to be separated. The genital double-somite of *H. cyanus* sp. nov., characterized by prominent lateral expansions directed posterolaterally, differs from that of all other congeners.

*Hemicyclops cyanus* sp. nov. also differs in the structure of the female leg 5: the exopodal segment is flexed and elongated, with a length-to-width ratio of 3.37. Although several species in *Hemicyclops*, including *H. amplicaudatus* Humes, 1965, have an elongated exopodal segment of leg 5, the segment in these species is straight, in contrast to the flexed condition in the new species.

Notably, the third endopodal segment of leg 3 of *H. cyanus* sp. nov. bears four spines and two setae (formula I, II, I+2, rather than I, II, 3 as usual), an unusual armature formula within *Hemicyclops* shared by only four species of the genus (*H. apiculus*, *H. australis*, *H. parapiculus*, and *H. intermedius*). All five of these species belong to Group II, and their caudal rami are short, measuring no more than 1.15 times as long as wide (Table 1).

The spermatophore is rarely reported in *Hemicyclops*, and the known forms vary among species. For example, it is elongated and domino-shaped in *H. parilis* Moon & Kim, 2010 and *H. gomsoensis* Ho & Kim, 1991, as illustrated by Moon and Kim (2010), and bulbous in *H. thysanotus* Wilson, 1935, as figured by Gooding (1960). The spermatophore of *H. cyanus* sp. nov. results from the fusion of a pair of spermatophores, with wing-like lateral extensions and a central longitudinal tube into which the urosome inserts. This morphology clearly distinguishes the species from all known congeners.

### ﻿Insufficiently described species

1. Species inquirendae: *Hemicyclops canuensis* (Bourne, 1890), *H. dilatatus* Shen & Bai, 1956, *H. indicus* Sewell, 1949, and *H. puffini* (Thompson, 1888). These four species were described solely on the basis of juveniles, and Vervoort and Ramirez (1966) already recognized them as insufficiently known. Diagnostic characters of *Hemicyclops* include the shape of the genital double-somite (Fig. 4), the proportional size of the caudal ramus, and the setation of the antennule and male maxilliped (Table 1). However, juveniles of *Hemicyclops* do not display these features. For example, *H. japonicus* Itoh & Nishida, 1993 has a complicated genital double-somite in the adult female (Itoh and Nishida 1993). Nevertheless, copepodid V of this species has a simple, subquadrate genital somite in both sexes (Itoh and Nishida 1995). The caudal ramus in copepodid V of the same species is 1.4 times as long as wide in both sexes (Itoh and Nishida 1995). Still, in the adult stage, the ramus is significantly longer, measuring 1.7 times as long as wide in females and 1.9 times as long as wide in males (Itoh and Nishida 1993). The setation of the antennule may not be complete until the adult stage, as shown in *H. ctenidis* Ho & Kim, 1990 (Kim and Ho 1992). Sexual differentiation of the maxilliped in *Hemicyclops* only appears at the adult stage. In *H. ctenidis*, a significant change in body length occurs between copepodid V and the adult stage (Kim and Ho 1992). These facts indicate that species of *Hemicyclops* should not be described based on juveniles. We recognize the four species mentioned above as species inquirendae.
2. Species with incorrect generic position: *H. dubia* (Thompson & Scott A, 1903) — this species does not belong to *Hemicyclops*. Thompson and Scott (1903) described this species based on a male collected in the Suez Canal. As described or figured in the original description of this species, the body is narrow, harpacticiform, the antennule is six-segmented, the maxillule is unilobed, the syncoxa of the maxilla lacks any armature element, the syncoxa of the male maxilliped bears a strong inner protrusion, and the third endopodal segment of leg 1 is armed with two spines plus four setae. Because these are diagnostic features of the genus *Conchyliurus* Bocquet & Stock, 1957, *H. dubia* should be removed to *Conchyliurus*. However, we would not decide here whether this is a definite species or a species inquirenda within *Conchyliurus*.
3. Species described from males: *H. javaensis* Mulyadi, 2005, *H. minutus* Mulyadi, 2005, and *H. leggii* (Thompson & Scott A, 1903). These three species were described entirely from males. Since the species of *Hemicyclops* have been described primarily based on females, the males of the above species cannot be reliably compared with existing species, because the morphological characteristics of the various appendages of the male are different from those of the female. Although these three species are likely to be treated as species inquirendae in the future, *H. cyanus* sp. nov. can be readily distinguished from the three species, since *H. javaensis* and *H. leggii* have one spine plus four setae on the third endopodal segment of leg 1 (formula I, 4; the only other species of *Hemicyclops* bearing this armature condition is *H. sebastiani* Kihara & Rocha, 1993, compared to the formula I, 5 in all other species in the genus), and *H. minutus* bears five (not four) setae on the first segment of the antennule.

*Hemicyclops cyanus* sp. nov. is distinguished from congeners by: (1) a genital double-somite with prominent posterolateral expansions; (2) a flexed, elongate exopodal segment of leg 5 (length-to-width ratio 3.37); and (3) a fused spermatophore complex with wing-like lateral extensions and a central tube. This is the first record of *Hemicyclops* associated with the coral family Euphylliidae, expanding the genus’s known host range and shedding light on its ecological associations.

## Supplementary Material

XML Treatment for Hemicyclops cyanus

## ﻿Additional information

### Use of AI

No use of AI was reported.

### Author ORCIDs

Il-Hoi Kim https://orcid.org/0000-0002-7332-0043

Viatcheslav N. Ivanenko https://orcid.org/0000-0003-1255-0491

### Data availability

All of the data that support the findings of this study are available in the main text or Supplementary Information.

## Supplementary materials

Supplementary material 1 Additional information Data type pdf Explanation note **fig. S1.** Underwater photograph of the host coral *Galaxea fascicularis* prior to sampling. **fig. S2.** Underwater photograph of the dive computer at the sampling site. **fig. S3.** Photograph of the skeleton of the host coral *Galaxea fascicularis*. **fig. S4.** Macrophotograph of the skeleton of the host coral. **fig. S5.** Map of the atoll showing the sampling location (red mark), Google Maps. https://binary.pensoft.net/file/1468295This dataset is made available under the Open Database License (http://opendatacommons.org/licenses/odbl/1.0/). The Open Database License (ODbL) is a license agreement intended to allow users to freely share, modify, and use this Dataset while maintaining this same freedom for others, provided that the original source and author(s) are credited.Il-Hoi Kim, Jia Wang, Viatcheslav N. Ivanenko
