# Supplementary material for: ﻿A new Hemicyclops (Copepoda, Cyclopoida, Clausidiidae) associated with the scleractinian coral Galaxea from the South China Sea
Source: Zookeys. 2025 Nov 18;1260:93–109. doi: 10.3897/zookeys.1260.168539 (PMC12648167; doi:10.3897/zookeys.1260.168539)
Supplement: Supplementary material 1 — Additional information [file zookeys-1260-093_article-168539__-s001.pdf]

Supplementary Figures 1-5

Figs 1–4 photographed by V.N. Ivanenko

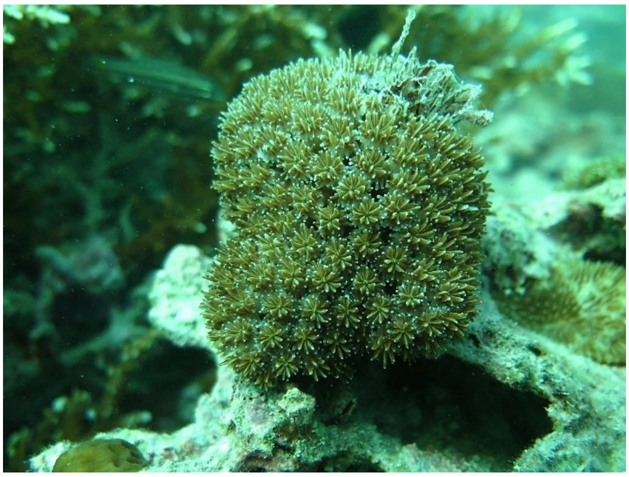

Fig. 1 – underwater photograph of the host coral *Galaxea fascicularis* prior to sampling

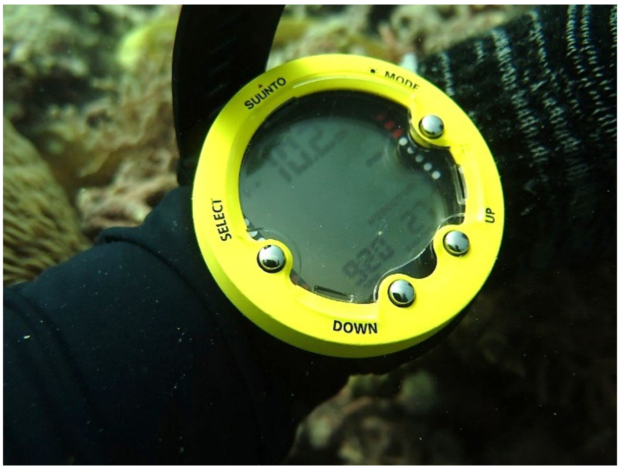

Fig. 2 – underwater photograph of the dive computer at the sampling site

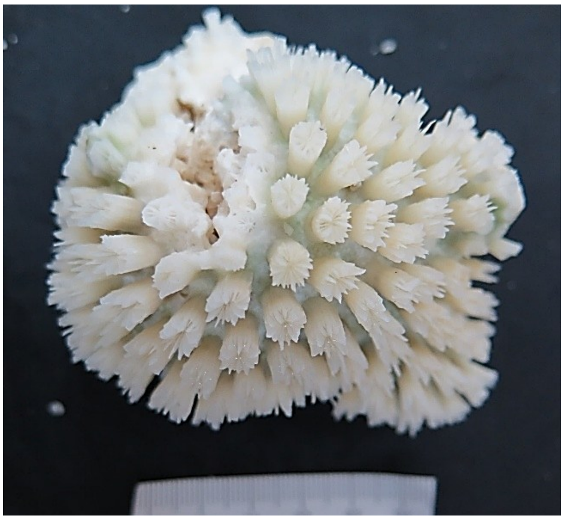

Figs 3 – photograph of the skeleton of the host coral *Galaxea fascicularis*

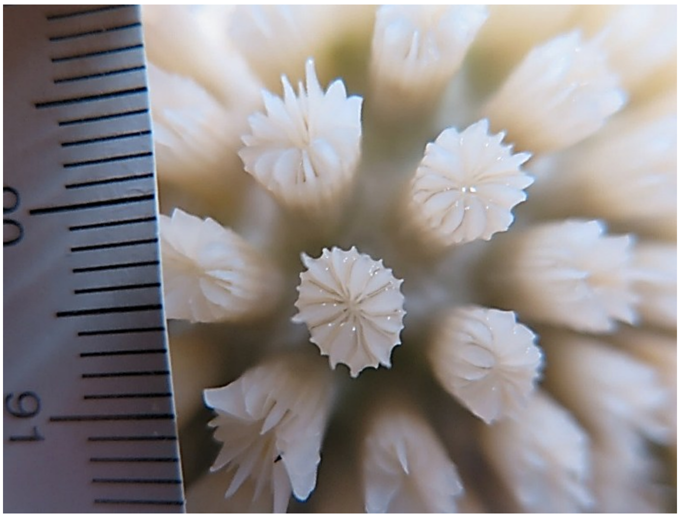

Figs 4 – macrophotograph of the skeleton of the host coral

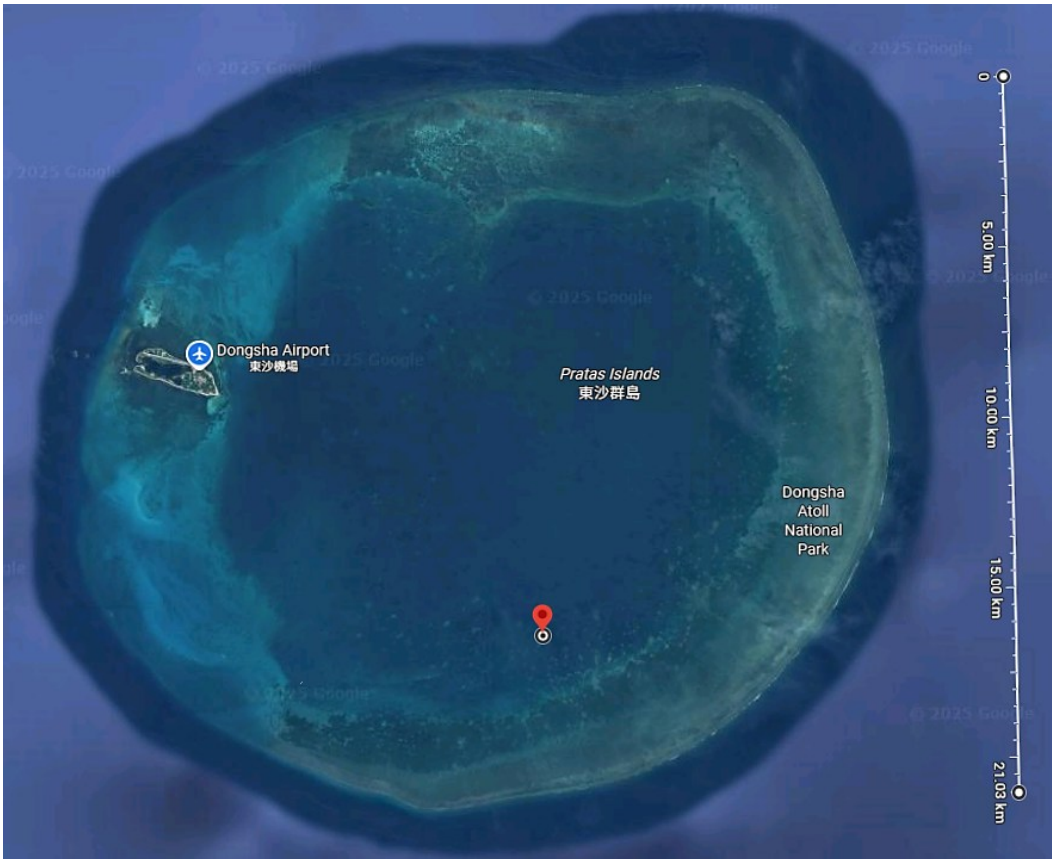

Fig. 5 – map of the atoll showing the sampling location (red mark), from Google Maps.
